# A Hospital for the Insane

**Published:** 1902-02-22

**Authors:** 


### A Hospital for the Insane.

A reform that was foreshadowed more than 12 years ago in London, but which could not then be carried into operation, has recently been advocated in Edinburgh, and seems likely to be adopted there. The members of the medical profession in that city have urged upon the authorities of the Royal Infirmary the desirableness of appropriating two of its wards for the reception and treatment of the insane, and their representations have received favourable consideration. It has long been the opinion of many observers that the knowledge of the nature and causes of insanity, and of the methods which might be adopted for its curative or preventive treatment, have lagged far in the rear of the progress which has been made in relation to other diseases of the nervous system, and that the reason of the difference has, at least to some extent, depended upon the fact that the conditions of a lunatic asylum have been eminently unfavourable to the prosecution of sustained clinical and pathological research. In April, 1889, Mr. Brudenell Carter, who at that time was a member of the London County Council, moved therein, "That a committee be appointed to inquire into, and to report to the Council upon, the advantages which might be expected from the establishment, as a complement to the existing asylum system, of a hospital with a visiting medical staff, for the study and curative treatment of insanity." The resolution was carried; and the Committee appointed in accordance with it held many meetings, examined a large number of medical witnesses of eminent position, addressed a circular of inquiries to asylum officers, and finally, on January 30th, 1890, unanimously reported to the Council, at the close of an explanatory document of some length :—

(*a*) That in the opinion of the most eminent and most experienced members of the medical profession, the knowledge which is possessed, with regard to the nature, prevention, and cure, of the diseased changes which underlie and occasion insanity, is not commensurate with that which is possessed with regard to diseased changes of other kinds, even those which affect other portions and other functions of the nervous system.

(*b*) That the difference in question is mainly due to the circumstance that patients suffering from insanity have been to a great extent withdrawn from the operation of the ordinary methods of hospital investigation and treatment, which have been so fruitful of good in the case of diseases of other kinds.

(*c*) That the establishment, on the ordinary lines, of a hospital for the study and treatment of insanity, with a visiting medical and surgical staff, could scarcely fail to be productive of increased knowledge of the subject, and, consequently, of increased means of prevention and cure.

The committee therefore recommended :

That an adequately equipped hospital, containing 100 beds, for the study and curative treatment of insanity in pauper lunatics of both sexes, be established in the metropolis under the direction and control of the council, and that its staff and general organisation should be in accordance with the principles laid down in the foregoing report.

This recommendation, in due course, was brought before the whole body of the Council, but only to be rejected by them. The fate of any recommendation, in those days, was determined in a secret meeting of the junta which controlled the majority; and hence there was scarcely even a semblance of any public discussion of the grave questions which were at issue in relation to this one. Each individual "progressist" had been told how to vote, and neither the supporters nor the opponents of the scheme had any inclination to spend their breath in argument about a proposal which they knew to be foredoomed. The report had, nevertheless, the one good consequence of directing the attention of the Asylums Committee of the Council to a grave defect in the organisations under their control, and hence it led to the appointment of a distinguished man of science as a "pathologist," although no step was taken to bring his work into touch with that of an equally distinguished clinical observer, without whom the office of the pathologist seems essentially to resemble that of the detective who calls attention to the particular door or window through which burglars had gained admission to premises which they had despoiled. We have revived this old story because it would appear, from what we have seen in print, that the good people of Edinburgh are somewhat pluming themselves upon occupying a position of originality in relation to the contemplated extension of the benefits of their infirmary. We desire to congratulate them most heartily upon being the first to give practical effect to the suggestion that insanity is a disease, which, like other diseases, can only be effectively studied and controlled by the aid of hospital organisation and resources, but at the same time we cannot refrain from placing on record for the credit of London. that this truth was recognised here 12 years ago. It was not acted upon for the reason that the elected of the ratepayers were not sufficiently intelligent to be able to appreciate the facts which were submitted to them. If the authorities of the Edinburgh Infirmary require any arguments in support of the course upon which they are understood to have determined, those arguments may be found in the published report made to the London County Council in January, 1890.

